# The General Factor of Personality as Ego-Resiliency

**DOI:** 10.3389/fpsyg.2021.741462

**Published:** 2021-11-22

**Authors:** Curtis S. Dunkel, Dimitri van der Linden, Tetsuya Kawamoto, Atsushi Oshio

**Affiliations:** ^1^Department of Psychology, Western Illinois University, Macomb, IL, United States; ^2^Department of Psychology, Education, and Child Studies, Erasmus University Rotterdam, Rotterdam, Netherlands; ^3^Faculty of Letters, Kokushikan University, Tokyo, Japan; ^4^Faculty of Letters, Arts and Sciences, Waseda University, Tokyo, Japan

**Keywords:** resileincy, ego adaptability, personality structure, structural equation (SEM), longitudinal

## Abstract

It was originally hypothesized by Block that what has come to be known as the General Factor of Personality (GFP) reflects ego-resiliency. We test Block’s hypothesis in two studies. In Study 1 a meta-analysis (*N* = 15,609) examining the relationship between the GFP and ego-resiliency/resilience was conducted. In Study 2 (*N* = 157) archival data from Block and Block was used to examine the association between rater judged ego-resiliency across childhood, adolescence, and into early adulthood and the GFP based on self-report in early adulthood. Using structural equation modeling for the meta-analytic data, the correlation between the GFP and ego-resiliency/resilience was estimated at *r* = 0.93. Using a trait-state occasion model to test the hypothesis in Study 2, the correlation between the GFP and rated ego-resiliency was estimated at *r* = 0.85. The results of the two studies offer substantial support for Block’s original hypothesis. Given the strength of the associations between the GFP and ego-resiliency/resilience one may conclude that the two constructs largely reflect the same underlying phenomenon.

## Introduction

The general factor of personality, or GFP, reflects the shared variance among personality traits in which individuals who possess one socially desirable characteristic are also more likely to possess another (Figueredo et al., 2004; Musek, 2007; Rushton et al., 2008). The chronological account of research on the GFP follows a narrative beginning with Galton’s (1884) recognition that although individual personality descriptors may have different shades of meaning, their connotations largely overlap along a negative to positive gradient. Webb (1915), however, is often credited with conducting the first formal empirical test of the GFP. He found a general factor in the personality ratings of groups of boys and young men. Webb (1915) represented the general factor with a lower case italicized *w* signifying “will” suggesting that it is comparable to *g* the general factor found when analyzing cognitive abilities.

Subsequent to Webb (1915), it is thought that research on the GFP remained dormant for close to a century. Meanwhile, Personality Psychology largely solidified around the Five Factor Model (e.g., McCrae and Costa, 1987) as the preferred paradigm for the structure of personality (Block, 1995). In the FFM, personality is largely defined by five higher-order and presumably orthogonal personality traits (Openness, Conscientiousness, Extraversion, Agreeableness, and Neuroticism). However, alternative models of personality structure remained (e.g., Ashton and Lee, 2007). Research on the possibility of a meaningful GFP was revived around 15 years ago (Figueredo et al., 2004; Musek, 2007). Initially, the scientific discussion focused on whether or not a general factor exists. However, the GFP has since then been confirmed numerous times in large individual studies with hundreds of thousands of participants (Erdle and Rushton, 2011; Dunkel et al., 2014), meta-analyses (Van der Linden et al., 2010, 2016), and extensive systematic reviews (Rushton and Irwing, 2011; Musek, 2017). With the establishment of the existence of a GFP, attention has been redirected at identifying its nature.

In light of this discussion on the nature of the GFP, here we focus on the work of Jack Block (e.g., Block, 1965, 2010; Block and Block, 1980a). Block was both an ardent critic of the emerging consensus around the FFM and a strong proponent of a person-centered and ego-based system to the study of personality. The personality system posited by Block includes two fundamental dimensions: ego-control and ego-resiliency. Ego-control is an individual’s modal response to internal urges and external distractions. An intermediate level of ego-control is associated with optimal psychosocial functioning. Too little ego-control results in impulsive behaviors and Block labeled individuals with low levels of ego-control undercontrollers. At the opposite end of the spectrum are overcontrollers who tend toward rigidity, restrictiveness, and fragility.

The other dimension of Block’s model, and the focus of the current paper, is ego-resiliency. Ego-resiliency represents the ability to adaptively modify the level of self-control to match the circumstances. Thus, it refers to flexibility in order to display adequate, context-specific behavior. An individual who is high in ego-resiliency is appropriately versatile. During a structured somber event such as a funeral they will increase control whilst at a casual after-hours get together they would loosen control. In each situation their behavior and actions would be fitting and proper; not boorishly undercontrolled, nor stiflingly overcontrolled. This description of ego-resiliency suggests strong overlap with other well-known constructs like emotional intelligence and social-effectiveness; a fact that Block often recognized (e.g., Block and Block, 1980a).

Block’s (1995, 2001, 2010) frequent negative critiques of the FFM are also suggestive of a GFP. Regarding this, we focus on three of his primary criticisms of the FFM that are relevant to the GFP concept. The first criticism is that the five traits are “*impressively non-orthogonal*” (1995, p.200). While Block’s criticism alludes to the possibility of a GFP, Block died in 2010 just as research into the GFP began to gather momentum (e.g., Van der Linden et al., 2010). Although in writings penned shortly before his death he consistently expressed enthusiasm for research on the Big Two higher-order meta-traits (Digman, 1997; DeYoung, 2006) and in a posthumous article included a footnote that referred to early research on the GFP. Thus, while not explicitly endorsing the GFP construct, Block recognized significant Big Five trait inter-correlations and the presence of higher-order personality factors.

The second criticism is that the factor structure is sample dependent (i.e., factor variance). Factor variance continues to be a problem for the FFM, yet a GFP consistently emerges across samples. A good example of this contrast is recent work with the Tsimane; an indigenous population located in Bolivia. Gurven et al. (2013) found that in this preliterate population, the FFM was a poor fit for underlying personality factor structure, yet consistent higher-order factors above the Big Five clearly emerged. Analyzing the same data, Van der Linden et al. (2018a) found a GFP using Tsimane participant’s self-reports and, furthermore, this GFP was validated with the positive impressions reported by others. These cross-cultural findings are not unique; a GFP has been confirmed in numerous Western and non-Western populations (e.g., Rushton et al., 2008; De Raad et al., 2010; Erdle and Aghababaeib, 2012; Dunkel, 2013; Wu et al., 2020).

The third of Block’s criticisms of the FFM we wish to highlight is that the FFM is atheoretical (i.e., it is not based in evolutionary theory). In contrast, the GFP is well-grounded in evolutionary theory (e.g., Figueredo et al., 2004, 2015; Rushton et al., 2008). A high GFP appears to provide linear fitness benefits. Individuals high in the GFP are more likely to secure social rewards and tangible resources (e.g., Wu et al., 2020). An example of a direct fitness advantage of a high GFP is found in the research on the Tsimane, the indigenous population of Bolivian introduced in the previous paragraph. Tsimane males with a higher GFP were found to have produced more offspring (Gurven et al., 2013; Van der Linden et al., 2018a), such that each standard deviation increase in the GFP is associated with a corresponding 0.88 increase in the number of children sired. While this reproductive advantage may push a population toward ever higher levels of the GFP, variation may result from mutation-selection balance (Verweij et al., 2012).

Though it is not vital that Jack Block explicitly speculated about a possible relationship between his ego-based model of personality and the GFP for a union to be hypothesized; such a statement would lend authority to the prediction. Additionally, an explicit statement would change the chronical account of research on the GFP described at the beginning of this manuscript. For these reasons this extended quote from his book on response sets in the Minnesota Multiphasic Personality Inventory (MMPI; Block, 1965) is presented. In this book Block discusses the meaning of the first factor extracted from the MMPI (referred to as Alpha) and contrasts his perspective, that this factor is substantive and meaningful, with the belief that this factor reflects error emanating from social-desirable response bias (SD).

“*If the first MMPI factor is not to be understood profitably in terms of the SD concept, just how should this dimension be construed in the light of the current analyses? Welsh’s [1956] earlier identification of this factor as “anxiety” would appear to be improved if changed to “susceptibility to anxiety.” Anxiety is a state of the individual where this factor reflects a characterological disposition. In other factor analyses of the MMPI, this repeatedly found first component has been variously identified as, for example, “psychoticism” [Wheeler et al., 1951] or “general maladjustment” [Tyler, 1951] and when reversed “social appropriateness” [Block and Bailey, 1955] or “ego strength” [Kassebaum et al., 1959]. None of these labels seems conceptually satisfactory when referenced to the behavioral correlates of this factor, as reported here. Psychotics may well place low on the Alpha dimension but other, clearly non-psychotic individuals also will be Alpha Lows. “General maladjustment” and “social appropriateness” perhaps are not incorrect as descriptive labels [for the general factor in the MMPI] but they are labels with no conceptual properties, no position within a theoretical framework from which predictions will flow. The term, “ego-strength,” is by now conceptually amorphous and used diversely, often simply as a jargonistic substitute for “adjustment.”*

*The reader has before him the data from which he can form his own understanding of the first MMPI factor. However, the writer cannot forego the opportunity of indicating his own current conception of the significance of this MMPI dimension.*

*It is suggested that this factor be identified as “ego-resiliency.” The word, resilient, implies the resourcefulness, adaptability, and engagement with this world that characterizes the individual placed high on this continuum; the word, ego, implies that an enduring, structural aspect of personality in involved. In conjunction, the term ego-resiliency, is intended to denote the individual’s characteristic adaptation capability when under the strain set by new environmental demands. Alpha Highs appear to react to the press of new and yet unmastered circumstances in resourceful, tenacious, but elastic ways and so may be termed ego-resilient. Alpha Lows, on the other hand, have small adaptive margins and consequently react to their stresses in rigidified or chaotic ways. Because they are not ego-resilient, they are unable to respond effectively to the dynamic requirements of their situation.*

*An individual who is unresilient will not be in a state of anxiety if the circumstances in which he functions are for him safe and predictable. Yet, it may be expected that, inevitably, an adaptively inelastic individual will find a wider range of environmental happenings to be disruptive of his personal economy, and distressing. Accordingly, he will present himself as more anxious, more maladjusted, less appropriate, less attuned to his world and, not least, as possessing personal attributes which society agrees undesirable.*

*Thus, the concept of ego-resiliency fits well the behavioral correlates of the first MMPI factor and can encompass the various interpretations previously offered of this dimension. The construct has the further advantages, moreover, of fitting into a theoretical framework of not being tied to a particular evaluative society or culture as a referent, and of predicting to additional and diverse environmental contexts; e.g., ego-resilient individuals, as measured by the MMPI, should relate-when relevant other variables are held constant- to both ability to resist distractions and the ability to associate in distant, even bizarre ways when instructed to do so (“regression in service to the ego”). For the several reasons, it is suggested that the first factor within the MMPI be identified as the Ego-Resiliency (ER) dimension.”* (pp. 110 – 112; Block, 1965).

Block clearly promoted the belief that the extracted general factor from the MMPI (i.e., the GFP) is not simply due to social desirability bias, but is substantive. At present, social-effectiveness has emerged as the preferred substantive account of the GFP (Dunkel and Van der Linden, 2014; Pelt et al., 2020), but Block appears to have had a slightly different opinion as to the nature of the GFP. While in the quote Block repeatedly references the strong association between social-effectiveness, emotional intelligence, and ego-resiliency he believes that the true relationship is one in which ego-resiliency encapsulates social-effectiveness and similar constructs leading Block to emphatically state that the first factor or GFP should be identified as ego-resiliency. To Block’s point, a strong relationship between the GFP and ego maturity has been found (Dunkel et al., 2011). However, to our knowledge the association between ego-resiliency, specifically, and the GFP has yet to be examined. In the present article, we will take this step by testing the ego-resiliency-GFP overlap in two studies. In the first study we reanalyzed meta-analytic data that has been collected to test the relationship between the Big Five and trait resilience and ego-resiliency^1^ (Oshio et al., 2018). The second study extends the first, by testing the relationship between the two constructs, measured over a period of 20 years.

The hypotheses in both studies are based on Block’s reasoning, which shows that he was (1) highly critical of the FFM, (2) believed that personality measures produced a GFP, (3) that this GFP was not due to social desirability, and (4) that this GFP reflected ego-resiliency. Thus, following this reasoning, the fundamental hypothesis to be tested is that the GFP will be positively associated with ego-resiliency. Furthermore, Block recognized the strong semantic and empirical associations between social-effectiveness, emotional intelligence, and ego-resiliency. Because the association between trait emotional intelligence and the GFP is strong enough to suggest unity between the two constructs (Van der Linden et al., 2017, 2018b) transitivity results in the additional prediction that the association between the GFP and ego-resiliency will also be strong (*r* > 0.50); possibly strong enough to suggest unity between the two constructs.

## Study 1: Meta-Analytic Data on Personality and Ego-Resiliency/Resilience

Although, to our knowledge, there are no previous studies that have directly tested the relationship between the GFP and ego-resilience, there have been several studies examining the association between the FFM traits and resilience – trait or ego. In fact, studies of the association between resilience and the FFM traits were recently subjected to a meta-analysis. This meta-analysis, conducted by Oshio et al. (2018), included 30 studies over a period ranging from 1997 to 2016, with a combined total of over 15,000 participants and although they didn’t examine the association between resilience and the GFP, the correlations of resilience with the FFM traits provides strong evidence that such an association exists. The correlations between resilience and the FFM traits were as follows: openness (*r* = 0.34); conscientiousness (*r* = 0.42); extraversion (*r* = 0.42); agreeableness (*r* = 0.31); neuroticism (*r* = −0.46), which is a pattern suggestive of the influence of the GFP. Accordingly, we reanalyze this meta-analytic data, such that a GFP can be extracted that can be directly related to resilience.

### Method

In order to test the relationship between the GFP and resilience we combined the results of two previous meta-analyses. The first is the study of Van der Linden et al. (2010) who used psychometric meta-analytic techniques to estimate the true intercorrelations between the dimensions in the FFM, Openness (O), Conscientiousness (C), Extraversion (E), Agreeableness (A), and Neuroticism (N). The results were based on all peer-reviewed internationally published articles between 2000 and 2008 that reported FFM intercorrelations, leading to total set of *K* = 212 matrices, representing a total of *N* = 414,117 participants. Approximately 67% of the studies in the meta-analysis had used the mainstream instruments such as the NEO Five Factor Inventory (NEO-FFI), the revised NEO (NEO-PI-R), the Big Five Inventory (BFI), or measures based on the International Personality Item Pool (IPIP). The remaining studies used less common, but validated questionnaires such as the Personal Characteristic Inventory (PCI), the Big Five Observer (BFO), Five-dimensional Temperament Inventory (FDTI), Trait Descriptive Adjective Scale (TDA), the Ten Item Personality Inventory (TIPI), and Hamburg Personality Inventory (HPI). The meta-analysis reported the weighted (by sample size) average correlations between the Five Factors, as well as the weighted averages, corrected for sampling error, unreliability, and range restriction. Meta-analytic values were calculated by entering the observed values in Excel sheets and applying the mathematical formulas as described in Hunter and Schmidt (2004).

In the present study we used these meta-analytic FFM intercorrelations because they are assumed to be more stable and reliable (i.e., reflect the true correlations) than the intercorrelations based on individual studies, or on meta-analytic studies with smaller *N*s. This notion is supported by the fact that in the van der Linden et al.’s meta-analysis, a variety of different types of samples (e.g., population, students, and adolescents) and FFM instruments (e.g., NEO-FFI, BFI, and IPIP) were used. Follow-up analyses showed that, despite slight changes in the values, the FFM intecorrelations were rather similar over type of sample and instrument used.

For the correlations between the FFM dimensions and resilience we used the meta-analysis of Oshio et al. (2018). These authors used the PsychInfo and EBSCOhost databases to search all articles until 2016 that reported zero-order correlations between FFM dimensions and resilience. With this procedure they identified 30 articles with a total *N* of 15,609. However, as different numbers of articles were found for different dimensions, the *N*s in the analyses on the Big Five and resilience ranged from 4,090 to 10,674. In Table 1 (p. 57) in their meta-analysis, Oshio et al. provide a list of the Big Five and resilience measures that were used in the studies they included. All instruments were well-known and validated instruments. For example, measures of resilience included the Dispositional Resilience scale, the Resilience Questionnaire, the California Adult Q-set, the Resilience scale, and the Wagnild and Young Resilience scale. The Five personality factors were measured with different validated scales, and included many of the well-known instruments such as the NEO (FFI or PI, PI-R), the BFI, IPIP, and 5-PFs. Oshio et al. reported the mean observed correlations, and the mean correlations corrected for unreliability. The data analysis conducted by Oshio et al. were done using Microsoft Excel in combination with the mathematical formulae of the random effects model (e.g., Borenstein et al., 2010). The values of the meta-analytic correlations between the FFM and resilience are also reported in Table 1.

**TABLE 1:** Meta-analytic correlations between the Big Five and ego-resilience as reported in Oshio et al. (2018).

FFM	*K*	*N*	Observed (mean) Correlation	ρ	*Q*
Openness	7	4090	0.46	0.50	19.72
Conscientiousness	7	4090	0.42	0.45	20.36
Extraversion	9	4732	0.44	0.47	30.14
Agreeableness	7	4090	0.39	0.42	17.39
Neuroticism	9	4732	–0.56	–0.63	18.52

*K, the number of studies included; N, number of participants; ρ, correlations corrected for unreliability; Q, Q-test of variability in effect sizes.*

Combining the meta-analytic values of Van der Linden et al. (2010) and Oshio et al. (2018) led to a matrix that included the FFM intercorrelations as well as their correlations with resilience. This matrix was the basis for subsequent structural equation modelling (SEM) in which we extracted a GFP from the Big Five, which was then related to resilience. In this procedure, decisions had to be made regarding the size of the *N* used in the matrix because the *N* differs for several of the values in matrix (see previous section). In line with previous studies, we decided to set the overall *N* equal to the lowest *N* in the two meta-analysis. In this case this was 4090. Although this decision implies that we tested relatively conservative, this was not a problem as the smallest *N* was still more than sufficient to detect even small relevant effect sizes.

In the original study of Van der Linden et al. (2010) it was already shown that a viable GFP can be extracted for the FFM intercorrelation matrix and that the model with the best fit was a hierarchical model in which the GFP directly loaded on two intermediate higher-order factors labeled stability and plasticity (DeYoung, 2006). Stability loaded positively on Conscientiousness, Agreeableness, and negatively on Neuroticism. Plasticity loaded on Openness and Extraversion. In the present study, the same model also served as the basis for examining the relation between the GFP and resilience, although we also tested alternative models for comparisons. In evaluating the model fits we used the criteria as described in Hu and Bentler (1999).

### Results

**FIGURE 1:**
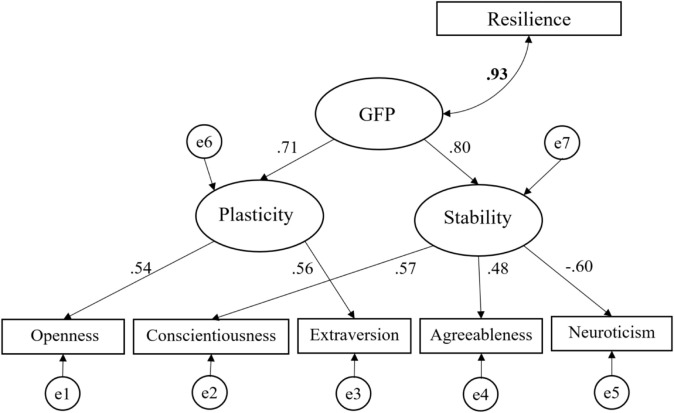
The hierarchical GFP model and its association with resilience.

Figure 1 shows the main model we tested. This model had a very good fit according to all of the indices we used (see Table 2). Obviously, the model reveals a viable GFP as this was already shown in the original meta-analysis (Van der Linden et al., 2010). The GFP loaded highly on Stability and Plasticity, which subsequently loaded highly on C, A, and N-, and O, and E, respectively. Most relevant for the present study was that in this model, in which we added resilience, the correlation between the GFP and resilience was so strong, *r* = 0.93 (C.I. = 0.928 −0.932), that they could be considered nearly identical. Additionally, because there were two studies that showed relatively extreme values on the relationships between the Big Five and ego resilience, we also calculated the relationships excluding these two studies. However, we found that doing so had a negligible effect on the overall results (see Supplementary Figure 1). Therefore, we decided to retain the studies in the analysis, in line with the notion that a meta-analysis should include all published studies.

**TABLE 2:** Fit indices for different models.

Model	χ^2^	*df*	*p*	CFI	TLI	RMSEA	AIC	BIC
Hierarchical GFP model	235.63	9	<0.001	0.97	0.96	0.05	259.63	344.34
Direct GFP model	600.47	9	<0.001	0.93	0.88	0.09	624.47	709.23
Direct GFP with corr. errors	263.27	7	<0.001	0.97	0.94	0.07	291.27	390.15
Independent five factors	3869.53	10	<0.001	0.54	0.31	0.21	3918.53	3996.23

Although the initial model we tested was effectively described the data, we still wanted to examine whether there were alternative models with a better fit. However, as is shown in Table 2 all these alternatives showed a worse fit than the main model. For example, a model in which we omitted the two intermediate higher-order factors and allowed resilience to correlate to a GFP that directly loaded on the FFM dimensions, had a much higher chi-square value and showed an acceptable fit on some indices, but not on others (Table 2). Allowing two unique variances to correlate in this latter model (based on modification indices) improved the fit, but it still was significantly worse than the initial, hierarchical, GFP model (Δχ^2^ = 27.64, Δ*df* = 2, <0.001). A model in which we assumed independent FFM dimensions (the basic theoretical assumption in the FFM) that each correlate with resilience, showed a very poor fit. This model could not be satisfactory improved by adding a few correlations in the FFM.

### Discussion

Using the values found in two large meta-analyses, the present study confirmed the predictions of Block (1965) that the GFP is indicative of resilience. The correlation that we found in the model is so high that, given inherent measurement error, they can be considered to be identical. We will elaborate on the theoretical implications of this overlap in the section “General Discussion”.

However, in brief, because the findings in Study 1 are based on many independently conducted studies, by different research groups, with a diverse population of participants, using a variety of measures, and conducted over several decades, the results provide strong and convincing support for the GFP-resilience overlap. Nevertheless, the study is not without limitations. In the vast majority of studies included in the meta-analysis, both resilience and the FFM personality traits were measured using self-reports that utilized Likert-type response scales. It has been shown that the GFP likely reflects method artifact as well as substantive variance (Dunkel et al., 2016). Therefore, the shared method variance across the two constructs is also likely inflated implying that the “true” shared variance between resilience and the GFP might be significantly lower than the 0.93 value reported. In Study 2 this limitation is addressed by using mixed methods, both in terms of the manner in which the individual differences are assessed (i.e., self and other ratings) and the type of scale utilized to score the rating. Additionally, the data in Study 2 includes numerous assessments of ego-resiliency across participants’ first two decades of life. This allows us to examine the association between the GFP and an individual’s pervasive form of ego-resiliency over a 20 year time span and several waves of data collection.

## Study 2: Association Between the GFP and Ego-Resiliency Across Time

### Method

#### Participants

Archived data from the Block and Block (2006a) longitudinal study was used. The Block and Block study involved extensive psychological testing of participants over a 30 year period, beginning when the participants were 3 to 4 years of age (Block and Block, 2006b). The data used in the current analyses were drawn from collection waves at age’s three to four, seven, 11, 14, 18, and 23. In these data collection waves the relevant personality measures were administered. One hundred and fifty-seven participants had personality data for the first wave of data collection at age’s three to four (98 at age seven, 106 at age 11, 106 at age 14, and 104 at age 18). The sample was almost evenly split between males (78) and females (79). Additionally the ethnicity of the sample was recorded as 98 Whites, 48 Blacks, seven Asians, and three “other”.

### Measures

#### Ego-Resiliency

At each age, ego-resiliency was measured *via* Q-sorts. For ages three to four, seven, 11, and 14 the California Child Q-set (CCQ; Block and Block, 1980b) was used. For ages 18 and 23 the California Adult Q-set (CAQ; Block, 1978) was used. In order to derive an ego-resiliency score a template or prototype approach was used. For both the CCQ and CAQ a prototype for ego-resiliency is included in the Block and Block (2006a) documentation files.

The five CCQ-set items most representative of ego-resiliency are: *is vital, energetic, lively; is resourceful in initiating activities; is curious, eager to learn, open to new experiences; is self-reliant, confident, trust own judgment; can recoup or recover after stressful experience*. The five Q-set items least representative of ego-resiliency are: *is inappropriate in emotive behavior; tends to go to pieces under stress; becomes rigidly repetitive or immobilized under stress; appears to feel unworthy, thinks of self as “bad”; is inhibited and constricted*.

In creating the CCQ prototype for ego-resiliency, Funder et al. (1983) reported an interrater reliability, between the three personality psychologists sorting the CCQ items to create an ego-resiliency template, of 0.90. At the ages three to four, Q-sorts for each participant were created by six nursery school teachers. At age seven, Q-sorts were made by two examiners and one teacher. At age 11, Q-sorts were performed for each participant by four to five examiners. At age 14, four examiners created Q-sorts for each participant. The estimated internal consistency of the CCQ-items based on correlations among raters, as reported in the documentation files, is as follows: 0.65 at ages three and four; 0.47 at age seven, 0.63 at age 11, and 0.72 at age 14.

The five CAQ-set items most representative of ego-resiliency are: *has insight into own motives and behavior; has warmth, capacity for close relationships; has social poise and presence; is productive, gets things done; calm, relaxed in manner*. The five Q-set items least representative of ego-resiliency are: *brittle ego-defense, maladaptive under stress; is self-defeating; is uncomfortable with uncertainty and complexities; over-reactive to minor frustrations, irritable; denies unpleasant thoughts and experiences*.

At age 18, four assessors and two interviewers Q-sorted the participants. At age 23, three assessors and two interviewers made the Q-sorts for each participant. Funder and Block (1989) reported an interrater reliability of 0.97 among the nine personality psychologists who were tasked with the job of creating a CAQ ego-resiliency template. The estimated internal consistency of the CAQ-items, as reported by Block and Kremen (1996), is 0.59.

The overall GFP in this study was based on the general factors extracted from three self-report measures of the Big Five personality traits of openness, conscientiousness, extraversion, agreeableness, and neuroticism (or emotional stability depending on the measure) that were administered at age 23.

One GFP was derived from the CAQ measure of the Big Five traits. Each participant completed a self-sort (their view of their own personality) using the CAQ. Big Five trait scores for the self-sort were tabulated using the method for transforming a CAQ sort into the Big Five from McCrae et al. (1986). The internal consistency for the trait scales were as follows: openness (α = 0.59), conscientious (α = 0.43), extraversion (α = 0.79), agreeable (α = 0.77), and neuroticism (α = 0.89).

The second GFP was also derived from another participant self-sort. In this self-sort participants rated their own personality using the 43-item Adjective Q-set. In this sort, items were arranged in a seven column quasi normal distribution reflecting the degree to which each item was representative of the participant’s personality. Aguilar et al. (1998) outlined how the Adjective Q-sort can be used to measure the Big Five traits. Using the instructions of Aguilar et al. (1998) following internal consistency for each trait score was as follows: openness (α = 0.48), conscientiousness (α = 0.73), extraversion (α = 0.59), agreeableness (α = 0.46), and neuroticism (α = 0.64).

The third Big Five measure was the NEO-FFI form S (Costa and McCrae, 1988) which is a 60-item self-report scale using a five-point Likert-type rating scale. The subscales representing each of the Big Five had the following internal consistencies; openness (α = 0.79), conscientiousness (α = 0.79), extraversion (α = 0.80), agreeableness (α = 0.80), and neuroticism (α = 0.87). A composite GFP was created by standardizing each of the GFP values and summing the *z*-scores.

In the present study, we extracted the super-GFP factor from those three different GFPs because those three GFPs were mutually strongly correlated with each other (see Table 3). While, extracting a super GFP factor cannot account for possible common method bias related to self-report, nevertheless, such a super factor can account for possible bias related to the specific instrument of measurement.

**TABLE 3:** Correlation coefficients Study 2.

		1	2	3	4	5	6	7	8

**Ego-resiliency**									
1	Age 3 to 4								
2	Age 7	0.45 [0.27, 0.59]							
3	Age 11	0.26 [0.08, 0.43]	0.48 [0.29, 0.62]						
4	Age 14	0.09 [–0.11, 0.27]	0.44 [0.25, 0.59]	0.59 [0.44, 0.70]					
5	Age 18	0.17 [–0.03, 0.35]	0.39 [0.19, 0.56]	0.49 [0.33, 0.63]	0.55 [0.40, 0.67]				
6	Age 23	0.16 [–0.03, 0.35]	0.14 [–0.08, 0.34]	0.29 [0.10, 0.46]	0.40 [0.23, 0.55]	0.56 [0.41, 0.68]			
**GFP**									
7	GFP_*CAQ*_	0.03 [–0.17, 0.23]	0.18 [–0.04, 0.39]	0.16 [–0.04, 0.35]	0.41 [0.23, 0.57]	0.43 [0.26, 0.58]	0.52 [0.36, 0.66]		
8	GFP_*NEO–FFI*_	0.21 [0.01, 0.39]	0.26 [0.05, 0.45]	0.27 [0.07, 0.45]	0.38 [0.20, 0.54]	0.43 [0.25, 0.57]	0.49 [0.32, 0.63]	0.69 [0.56, 0.78]	
9	GFP_*adjective Q–sort*_	0.00 [–0.21, 0.20]	0.14 [–0.08, 0.35]	0.22 [0.02, 0.41]	0.46 [0.28, 0.60]	0.31 [0.11, 0.48]	0.35 [0.15, 0.51]	0.68 [0.55, 0.78]	0.70 [0.58, 0.79]

**FIGURE 2:**
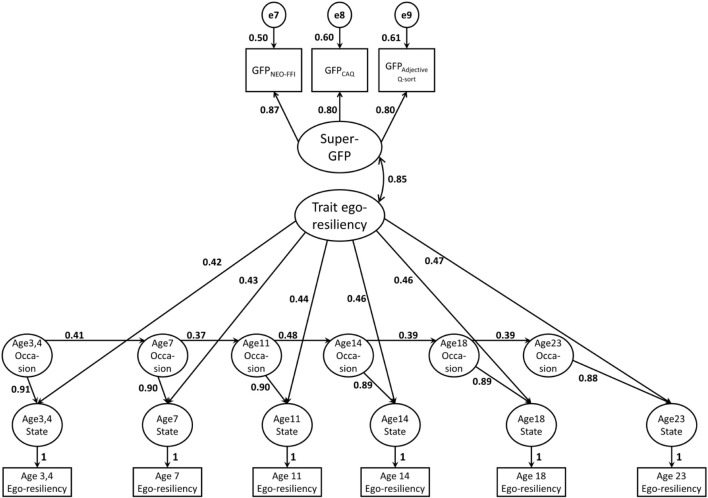
Standardized path estimates for the Trait-State-Occasion model.

In order to test the GFP-resilience relationship over a period of approximately 20 years, we used the trait-state occasion (TSO) model (Cole et al., 2005) in structural equation modeling. In the TSO model the state variance of ego-resiliency at each time point could be separated into two components: the trait factor and the occasion factor. The trait factor reflects a time-invariant component of ego-resiliency and the occasion factor in each time point reflects a time-varying component of ego-resiliency. The TSO model enables us to represent the relative stability of ego-resiliency over time and to investigate an association between the stable factor (trait) of ego-resiliency and the GFP. Regarding the GFP, we extracted the higher-order super-GFP factor from the three GFPs that were derived from the different personality measures. To achieve this, we combined the TSO model with confirmatory factor analysis model (see Figure 2) and applied it to the longitudinal data. Since the three GFPs and the last ego-resiliency score were measured at 23 years of age, the association between super-GFP and the stable trait factor of ego-resiliency might be inflated. In order to avoid over-evaluation of the association, we complementarily conducted the same analysis without the age 23 ego-resiliency score.

Because the data had some missing values we conducted Little’s missing completely at random (MCAR) test (Little, 1988). The MCAR test revealed that the null hypothesis of MCAR in the present data were not rejected (χ^2^(148) = 170.29, *p* = 0.10 for the full data; χ^2^(126) = 151.27, *p* = 0.06 for the data without age 23 ego-resiliency). Thus, we used full-information maximum likelihood method (FIML) to estimate the parameters. All statistical analyses were performed on R Statistical Software (ver. 4.0.1) using *lavaan* package (Rosseel, 2012) to conduct the structural equation modeling.

### Results

The correlations (and confidence intervals) between all variables are presented in Table 3. Regarding the ego-resiliency scores, we observed a simplex-like pattern. The rank-order stability between the two adjacent scores ranged from 0.45 to 0.59. As the measurement interval got longer, the rank-order stability was reduced. Correlations between ego-resiliency scores and the three GFPs were generally positive. Especially, the GFP from NEO-FFI was significantly correlated to all ego-resiliency scores (rs > 0.21).

The TSO model tested (χ^2^ = 53.08; *df* = 26; and *p* = 0.001) showed an acceptable fit to the data: CFI = 0.923, TLI = 0.893, RMSEA = 0.081, and SRMR = 0.097, though the TLI did not reach the criteria for good fit (Hu and Bentler, 1999). The standardized parameter estimates are shown in Figure 2 and their detailed confidence intervals are presented in Supplementary Table 2. The time-series regression parts of the occasion factors were positive and moderate. As for the confirmatory factor analysis component, the super-GFP factor loaded strongly on each of the three GFPs. In line with the main premise in this research, the super GFP-factor was positively and significantly associated with trait ego-resiliency factor (*r* = 0.85, 95%*CI* = [0.52, 1.00], and *p* < 0.001). In complemental analysis using the data without age 23 ego-resiliency, the TSO model showed an acceptable fit to this data (χ^2^(19) = 39.28, *p* = 0.004; *CFI* = 0.932, *TLI* = 0.900, *RMSEA* = 0.082, and *SRMR* = 0.092). The positive correlation between the super-GFP and trait ego-resiliency was lower but still substantial and statistically significant (*r* = 0.66, 95%*CI* = [0.38, 0.94], and *p* < 0.001). The standardized parameter estimates and their confidence intervals are presented in Supplementary Figure 2 and Supplementary Table 2, respectively.

### Discussion

Based on the 20-year longitudinal data, we investigated the association between ego-resiliency from childhood to adolescence, and the GFP in young adulthood. The simplex-like correlations among the six ego-resiliency scores strongly confirmed the relative stability of ego-resiliency over time. This was also supported in the trait-state-occasion model when extracting the time-invariant trait factor and regressing later occasion factor scores onto the same occasion scores at previous time points. Thus the bivariate correlations and more complicated analyses controlling for variance associated with each individual wave of data collection pointed a trait like ego-resiliency that exhibited substantial stability across time. Thus, the bivariate correlations and more complicated analyses controlling for variance associated with each individual wave of data collection pointed a trait like ego-resiliency that exhibited substantial stability across time.

The GFPs extracted from three different personality measures were substantially correlated with each other and could produce a reliable super-GFP factor, which is in line with the notion that a consistent GFP can be found in any measure that comprehensively assesses personality (e.g., Rushton and Irwing, 2011; Loehlin, 2012). The super-GFP factor was strongly positively associated with the time-invariant (trait) factor of ego-resiliency. This seems to suggest that the stable ego-resiliency component from childhood to adolescence plays a major role in developing, -or is largely similar to- the GFP in adulthood. This association remained significant after excluding the age 23 ego-resiliency from the longitudinal data. The latter finding shows that the ego resiliency-GFP overlap was not due to an inflated correlation between the ego-resiliency and the GFP measured at the same time. To illustrate, the simple bivariate correlations between ego-resiliency at age 23 and each GFP measured at the same age, ranged from 0.35 to 0.52. Yet the stable trait factor that was time-invariant from age 3 to 23 was much stronger related to the super-GFP (*r* = 0.85). By excluding the time-variant fluctuations in ego-resiliency, the findings show that the core component of social effectiveness has already been developed in relatively early developmental stages.

## General Discussion

The belief that there is a substantive primary personality trait (i.e., the GFP) reflecting the generalized range of biopsychosocial functioning may date to the very origins of recorded thinking on individual differences in personality (Figueredo et al., 2015). The account of the beginning of empirical research into this possibility is often credited to Galton (1884) or Webb (1915) with little subsequent interest or even awareness into a potential GFP until relatively recently (Figueredo et al., 2004; Musek, 2007).

However, this chronology is wrong. Mid-20th century personality researchers were engaged in an active and robust debate concerning the nature of what has now become to be known as the GFP. In fact, the debate over the first factor to emerge from the results of factor analyses of the MMPI is strikingly similar to the contemporary dispute over the GFP (Chang et al., 2012; Revelle and Wilt, 2013; Van der Linden et al., 2016, 2017; Ashton et al., 2020). Block (1965) argued for the meaningfulness of this first factor; maintaining that it reflected his concept of ego-resiliency. The alternative view was that the first factor was a result of measurement error such as response acquiescence (Messick and Jackson, 1961a) or social-desirability bias (Messick and Jackson, 1961b). The equivalence of Block’s position with the modern conceptualization of a GFP was tested in the current investigation. It was reasoned that if (1) Block believed that the first unrotated factor derived from the MMPI reflected ego-resiliency and (2) that the first unrotated factor derived from the MMPI is also the GFP (e.g., Rushton and Irwing, 2011) then ego-resiliency and the GFP exhibit a very strong association (if not unity).

This hypothesis was tested in two studies. In order to address method variance, the two studies employed disparate methods. In Study 1, the results of the meta-analysis of the association between the FFM traits and resilience were reanalyzed to test the possibility that the relationship between the FFM traits and resilience was largely a function of the GFP. In Study 2, we examined the associations between the stable Q-sort rater-assessed ego-resiliency throughout childhood and adolescence on the one hand, and the GFP in young adulthood on the other hand. The results of each study supported the main hypothesis that there is a strong association between the GFP and ego-resiliency.

In Study 1, the modeled association between the GFP and resilience was estimated at 0.93. In Study 2, that examined the stable components of resilience and the GFP over a period of 20 year, the correlation between the two constructs was found to be 0.85 (or 0.66 without age 23 ego-resiliency). The first finding strongly suggests that the GFP construct is quite similar with (or nearly equal to) ego-resiliency in adulthood. Additionally, the second finding strongly indicates that the GFP construct is developmentally related to the stable core component of ego-resiliency, which could extend the first finding. In summary the results of the two studies suggest an extensive overlap (or even unity) between ego-resiliency and the GFP.

We believe the results of the two current studies are of marked importance for two prospective reasons; firstly, conceptualizing the GFP as ego-resiliency leads to new insights and predictions. The ego acts to synthesize the self across time and space (Block, 1961; Erikson, 1968; Chandler et al., 2003), thus those high in ego-resiliency may also be more capable of integrating across personality characteristics as well, giving rise to a higher GFP (Chen et al., 2016). As such, those who are able to adaptively adjust to and incorporate life experiences may be in a better position to not only withstand life’s slings and arrows (Van der Linden et al., 2018c), but to exhibit positive growth and development from overcoming these stressors (Chen et al., 2016). These predictions, derived from the perspective of Positive Psychology (Gable and Haidt, 2005), are currently underexplored in the GFP literature. Likewise, the definition of the GFP as social-effectiveness may be too narrow (e.g., Smith et al., 2020). In contrast to the focus on social-relations, viewing the GFP as ego-resiliency leads to predictions on how the GFP may relate to internal cognitive states (Block and Kremen, 1996). High GFP (i.e., high ego-resiliency) individuals should be able to modify cognitive constraints appropriately; they may, for example, narrow cognitive focus to enhance concentration (e.g., flow) or alternatively loosening said constraints to uncover new and unique solutions (e.g., creativity). Nascent research in these areas has begun. Results have shown a positive association between flow propensity and the GFP (Ullén et al., 2016; Marty-Dugas and Smilek, 2019), while the results on the association between the GFP and creativity are equivocal (Lebuda et al., 2019; Rodriguez et al., 2020).

The jangle fallacy is when two nearly identical concepts are referred to by different names (Kelley, 1927; Block, 1995). The second reason we view the current findings as important relates to this persistent issue in the conceptualization and assessment of individual differences in personality. The GFP has shown extremely strong correlations with other general factors, namely the General Factor of Psychosocial Development (Dunkel et al., 2012), the General Factor of Psychopathology (Oltmanns et al., 2018), the General Factor of Personality Disorder (Oltmanns et al., 2018), the General Factor of Character (Tucker-Drob et al., 2016; Dunkel and Van der Linden, 2017) and trait emotional intelligence (Van der Linden et al., 2017, 2018b). Given the strength of the associations; the application of transitive reasoning leads to the conclusion that all of these concepts may reflect more or less the same underlying phenomenon. The results of the current investigation further suggest that ego-resiliency should be included in this list of unified constructs.

While we credit Block with the initial insight of GFP ≈ ego-resiliency, we are also not the first contemporary research team to make this connection. We argue that Gerlach et al. (2018) found strong evidence for this association without explicitly stating so. Using Block’s ego based personality theory as a theoretical framework; Gerlach et al. (2018) attempted to unearth a personality typology that lay within FFM scale scores. The analyses of data from over 1.5 million participants revealed four personality types one of which they labeled “role-model” which they, in turn, equate with ego-resiliency. The role-model type exhibits the same FFM social-desirable profile as the GFP: high in openness, conscientiousness, extraversion, and agreeableness while low in neuroticism. Thus, it may be more accurate to state the current findings provide further evidence for the synonymy between the GFP and ego-resiliency originally uncovered by Gerlach et al. (2018).

## Conclusion

In Block’s (1965, 1995) conceptualization, ego-resiliency represents the ability to adapt to the context in order to obtain one’s personal or communal goals (Irwing et al., 2020). As people are social by nature, such adaptation often implies displaying socially desirable (Schwartz et al., 1997) and appropriate behavior (e.g., being reserved during a funeral, being outgoing at a party). The ability to do so overlaps with labels such as social effectiveness, emotional intelligence, and self-regulation (Bar-On, 2000). In addition, effective adaptation to, for instance, social situations does not only require adequate display of behavior but also the regulation of one’s internal states (Pekaar et al., 2020). Such an ability to effectively adapt or regulate actions and internal states to the context is likely to have a broad effect on behavior: it can be expected to, at least partly, become manifest in many of the constructs measured in social science such as personality, self-confidence, social skills, emotional and cultural intelligence, grit, and many others. This may be the reason that, as Galton (1884) already noted, socially desirable or effective traits tend to go together (show a positive manifold). The present set of studies supports this notion and adds knowledge by showing that the resulting general factor also includes ego-resilience.

This notion has implications. If, as some scholars have suggested, the general factor would only reflect methodological or statistical bias, then it seems that many of the renowned constructs and research areas in social science may be largely based on artifact, and would thereby be, “mostly empty.” Yet, given the numerous studies showing the real-life relevance of concepts such as ego-resiliency, we consider the latter position not very plausible. We would prefer the interpretation that despite the myriad of possible differences between people, there are also general mechanisms driving behavior into a certain direction, thereby affecting a wide range of real-life outcomes such as job performance, social status, relationships, and citizenship (Van der Linden et al., 2016). As we already above, this interpretation or insight has the potential to unify research in by revealing the overlap between many key concepts in social science. However, it should also be noted that the results of these two studies are bound by the time and space in which the data was collected. As such it may prove fruitful to test for replication using a more contemporary sample.

## Data Availability Statement

Publicly available datasets were analyzed in this study. This data can be found here: https://murray.harvard.edu/.

## Ethics Statement

Ethical review and approval was not required for the study on human participants in accordance with the local legislation and institutional requirements.

## Author Contributions

CD contributed to the conception and design of the study. CD and AO acquired the data. DL and TK performed the statistical analysis. CD wrote the first draft of the manuscript. CD, DL, and TK wrote sections of the manuscript. All authors contributed to manuscript revision, read, and approved the submitted version.

## Conflict of Interest

The authors declare that the research was conducted in the absence of any commercial or financial relationships that could be construed as a potential conflict of interest.

## Publisher’s Note

All claims expressed in this article are solely those of the authors and do not necessarily represent those of their affiliated organizations, or those of the publisher, the editors and the reviewers. Any product that may be evaluated in this article, or claim that may be made by its manufacturer, is not guaranteed or endorsed by the publisher.

## Footnotes

1 Studies in the meta-analysis included both measures of ego-resiliency and trait resilience. Thus when referring to Study 1 we use the more inclusive term resilience. In Study 2 ego-resiliency was specifically measured and thus we use the term ego-resiliency when referring to Study 2.
