# Supplementary material for: The General Factor of Personality as Ego-Resiliency
Source: Front Psychol. 2021 Nov 22;12:741462. doi: 10.3389/fpsyg.2021.741462 (PMC8645775; doi:10.3389/fpsyg.2021.741462)
Supplement: Supplementary file 1 [file Data_Sheet_1.docx]

**The General Factor of Personality as Ego-Resiliency**

Supplemental materials


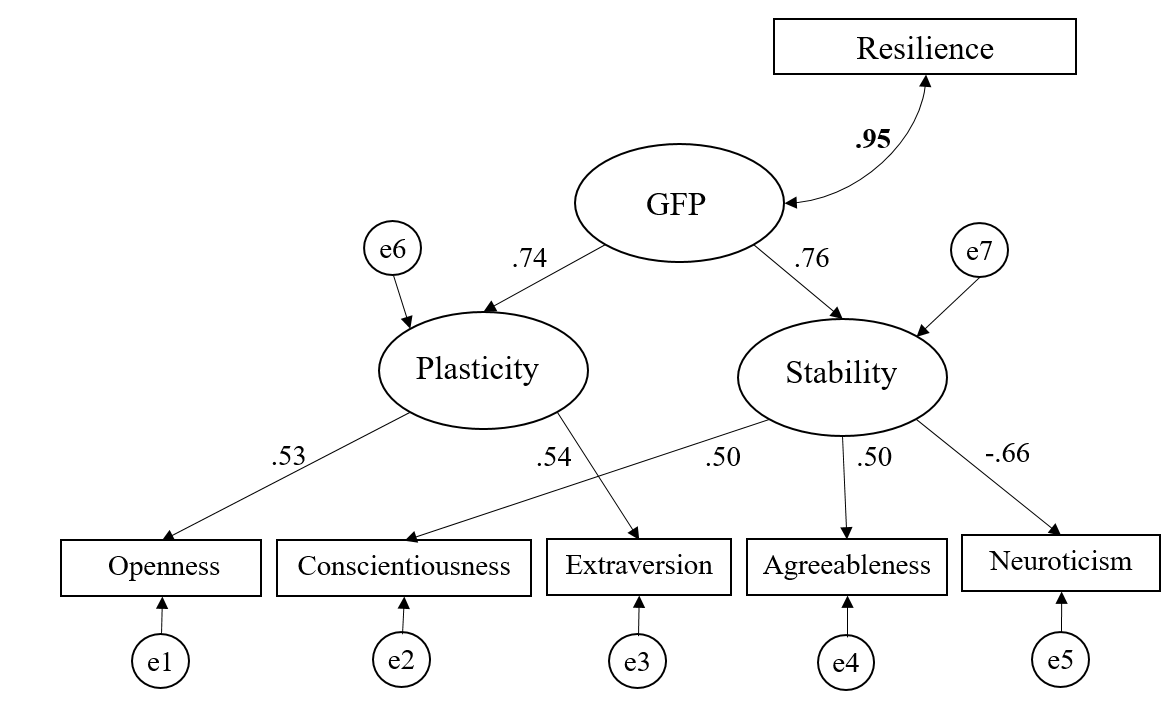


Figure S1. Model not including two outlying studies.

Table S1. Standardized parameter estimates and their 95% confidence intervals of the Trait-State-Occasion model.

|  | Parameters |  |  | *Estimates* | 95%CI | *p*-value |
| --- | --- | --- | --- | --- | --- | --- |
| Time series | |  |  |  |  |  |
| *β*_1_ | Age 3, 4 occasion | → | Age 7 occasion | 0.41 | [0.22, 0.60] | <0.001 |
| *β*_2_ | Age 7 occasion | → | Age 11 occasion | 0.37 | [0.16, 0.59] | 0.001 |
| *β*_3_ | Age 11 occasion | → | Age 14 occasion | 0.48 | [0.30, 0.66] | <0.001 |
| *β*_4_ | Age 14 occasion | → | Age 18 occasion | 0.39 | [0.18, 0.61] | <0.001 |
| *β*_5_ | Age 18 occasion | → | Age 23 occasion | 0.39 | [0.18, 0.60] | <0.001 |
| Trait factor loadings | |  |  |  |  |  |
| *λ*_1_ | Trait ego-resilience | → | Age 3, 4 state | 0.42 | [0.26, 0.59] | <0.001 |
| *λ*_2_ | Trait ego-resilience | → | Age 7 state | 0.43 | [0.26, 0.61] | 0.001 |
| *λ*_3_ | Trait ego-resilience | → | Age 11 state | 0.44 | [0.25, 0.62] | <0.001 |
| *λ*_4_ | Trait ego-resilience | → | Age 14 state | 0.46 | [0.27, 0.65] | <0.001 |
| *λ*_5_ | Trait ego-resilience | → | Age 18 state | 0.46 | [0.26, 0.66] | <0.001 |
| *λ*_6_ | Trait ego-resilience | → | Age 23 state | 0.47 | [0.28, 0.66] | <0.001 |
| Confirmatory factor analysis | | | |  |  |  |
| *λ*_7_ | Super-GFP | → | GFP_NEO-FFI_ | 0.87 | [0.78, 0.95] | <0.001 |
| *λ*_8_ | Super-GFP | → | GFP_CAQ_ | 0.80 | [0.70, 0.90] | <0.001 |
| *λ*_9_ | Super-GFP | → | GFP_adjective Q-sort_ | 0.80 | [0.70, 0.89] | <0.001 |
| Correlation | |  |  |  |  |  |
| *Cor* | Trait ego-resiliency | ↔ | Super-GFP | 0.85 | [0.52, 1.00] | <0.001 |


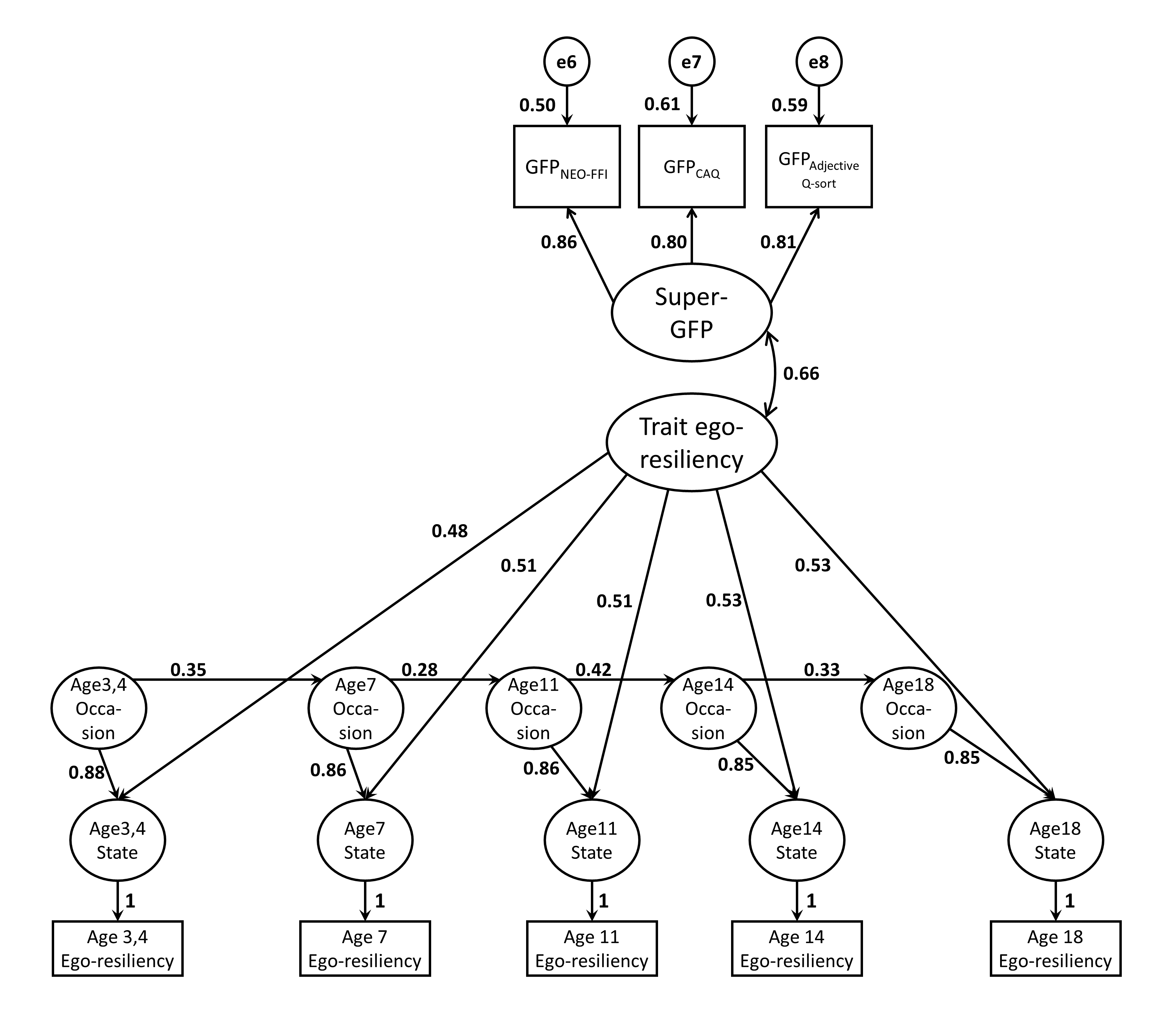


Figure S2. Standardized path estimates for the Trait-State-Occasion model without age 23 ego-resiliency.

Table S2. Standardized parameter estimates and their 95% confidence intervals of the Trait-State-Occasion model without age 23 ego-resiliency.

|  | Parameters |  |  | *Estimates* | 95%CI | *p*-value |
| --- | --- | --- | --- | --- | --- | --- |
| Time series | |  |  |  |  |  |
| *β*_1_ | Age 3, 4 occasion | → | Age 7 occasion | 0.35 | [0.13, 0.56] | 0.001 |
| *β*_2_ | Age 7 occasion | → | Age 11 occasion | 0.28 | [0.01, 0.54] | 0.039 |
| *β*_3_ | Age 11 occasion | → | Age 14 occasion | 0.42 | [0.21, 0.63] | <0.001 |
| *β*_4_ | Age 14 occasion | → | Age 18 occasion | 0.33 | [0.10, 0.57] | 0.006 |
| Trait factor loadings | |  |  |  |  |  |
| *λ*_1_ | Trait ego-resilience | → | Age 3, 4 state | 0.48 | [0.33, 0.64] | <0.001 |
| *λ*_2_ | Trait ego-resilience | → | Age 7 state | 0.51 | [0.33, 0.68] | <0.001 |
| *λ*_3_ | Trait ego-resilience | → | Age 11 state | 0.51 | [0.33, 0.69] | <0.001 |
| *λ*_4_ | Trait ego-resilience | → | Age 14 state | 0.53 | [0.35, 0.70] | <0.001 |
| *λ*_5_ | Trait ego-resilience | → | Age 18 state | 0.53 | [0.35, 0.72] | <0.001 |
| Confirmatory factor analysis | | | |  |  |  |
| *λ*_6_ | Super-GFP | → | GFP_NEO-FFI_ | 0.86 | [0.78, 0.95] | <0.001 |
| *λ*_7_ | Super-GFP | → | GFP_CAQ_ | 0.80 | [0.70, 0.89] | <0.001 |
| *λ*_8_ | Super-GFP | → | GFP_adjective Q-sort_ | 0.81 | [0.71, 0.90] | <0.001 |
| Correlation | |  |  |  |  |  |
| *Cor* | Trait ego-resiliency | ↔ | Super-GFP | 0.66 | [0.38, 0.94] | <0.001 |
